# Analytical validation of the Percepta genomic sequencing classifier; an RNA next generation sequencing assay for the assessment of Lung Cancer risk of suspicious pulmonary nodules

**DOI:** 10.1186/s12885-021-08130-x

**Published:** 2021-04-13

**Authors:** Marla K. Johnson, Shuyang Wu, Daniel G. Pankratz, Grazyna Fedorowicz, Jessica Anderson, Jie Ding, Mei Wong, Manqiu Cao, Joshua Babiarz, Lori Lofaro, P. Sean Walsh, Giulia C. Kennedy, Jing Huang

**Affiliations:** Veracyte, Inc., South San Francisco, CA 94080 USA

**Keywords:** Percepta, Genomic sequencing classifier, Molecular diagnostic, Lung lesion, Bronchial brushing specimen, Analytical validation

## Abstract

**Background:**

Bronchoscopy is a common procedure used for evaluation of suspicious lung nodules, but the low diagnostic sensitivity of bronchoscopy often results in inconclusive results and delays in treatment. Percepta Genomic Sequencing Classifier (GSC) was developed to assist with patient management in cases where bronchoscopy is inconclusive. Studies have shown that exposure to tobacco smoke alters gene expression in airway epithelial cells in a way that indicates an increased risk of developing lung cancer. Percepta GSC leverages this idea of a molecular “field of injury” from smoking and was developed using RNA sequencing data generated from lung bronchial brushings of the upper airway. A Percepta GSC score is calculated from an ensemble of machine learning algorithms utilizing clinical and genomic features and is used to refine a patient’s risk stratification.

**Methods:**

The objective of the analysis described and reported here is to validate the analytical performance of Percepta GSC. Analytical performance studies characterized the sensitivity of Percepta GSC test results to input RNA quantity, the potentially interfering agents of blood and genomic DNA, and the reproducibility of test results within and between processing runs and between laboratories.

**Results:**

Varying the amount of input RNA into the assay across a nominal range had no significant impact on Percepta GSC classifier results. Bronchial brushing RNA contaminated with up to 10% genomic DNA by nucleic acid mass also showed no significant difference on classifier results. The addition of blood RNA, a potential contaminant in the bronchial brushing sample, caused no change to classifier results at up to 11% contamination by RNA proportion. Percepta GSC scores were reproducible between runs, within runs, and between laboratories, varying within less than 4% of the total score range (standard deviation of 0.169 for scores on 4.57 scale).

**Conclusions:**

The analytical sensitivity, analytical specificity, and reproducibility of Percepta GSC laboratory results were successfully demonstrated under conditions of expected day to day variation in testing. Percepta GSC test results are analytically robust and suitable for routine clinical use.

**Supplementary Information:**

The online version contains supplementary material available at 10.1186/s12885-021-08130-x.

## Background

Lung cancer is the second most common type of cancer and the leading cause of cancer death in the United States, with 235,760 new cases and 131,880 deaths expected in 2021 [1]. Screening by low dose Computed Tomography (CT) has been shown to diagnose lung cancer at an earlier stage, resulting in improved prognosis and a relative decrease in mortality rates of 20%. However, the vast majority of nodules found by chest CT are benign, resulting in a high false positive rate of around 96% [2]. Currently, the American College of Chest Physicians (ACCP) and the National Cancer Comprehensive Network (NCCN) practice guidelines recommend an assessment of the risk of malignancy as the first step in lung nodule management. Nodules with a low risk of malignancy (< 10%) are recommended for radiological surveillance, while nodules with a high risk of malignancy (> 60%) are referred for surgical resection [3–5]. Patients with an intermediate risk of malignancy are often recommended to undergo a bronchoscopy in order to obtain a diagnosis. However, the diagnostic sensitivity of bronchoscopy varies considerably, based on a nodule’s location and size [6]. Nondiagnostic bronchoscopies may result in delayed diagnosis or unnecessary invasive procedures in patients with benign nodules.

The clinical management of suspicious lung nodules may benefit from the use of genomic tests [7]. Studies have shown that exposure to tobacco smoke alters gene expression in airway epithelial cells in a way that may increase the risk of developing lung cancer [8–10]. Gene expression profiling of epithelial cells collected during bronchoscopy could thus potentially improve the sensitivity of bronchoscopy for lung cancer detection [8–10]. Based on these ideas, a microarray-based clinical-genomic classifier, the Percepta Bronchial Genomic Classifier (BGC), was developed and validated in large, multicenter prospective trials in current and former smokers who had undergone bronchoscopy that did not return a pathology diagnosis [11, 12]. The primary clinical utility of the Percepta BGC was to reclassify the risk of malignancy in low and intermediate pre-test risk patients to low and very low post-test risk, respectively. In a clinical utility study, patients with down-classified post-test risk were more likely to undergo surveillance rather than an invasive procedure [13].

The Percepta Genomic Sequencing Classifier (GSC) is an RNA sequencing-based clinical-genomic risk stratification algorithm with additional clinical utility beyond that of the Percepta BGC. Similar to Percepta BGC, the main utility of Percepta GSC is in the down-classification of risk in low and intermediate pre-test risk samples. In a prospective clinical validation, the Percepta GSC down-classified low and intermediate pre-test risk patients with a sensitivity of 91% (95% CI 81–97%) and a specificity of 45% (95% CI 37–53%), resulting in a negative predictive value of 95% (95% CI 89–98%). The Percepta GSC can also accurately reclassify intermediate and high pre-test risk patients to high and very high post-test risk, respectively. Twelve percent of intermediate pre-test risk patients were reclassified as having high post-test risk of malignancy, with a positive predictive value of 65% (95% CI 44–82%). Additionally, 27% of high pre-test risk patients were reclassified to very high post-test risk with a positive predictive value of 91% (95% CI 78–97%) [14]. The clinical utility of up-classification of patients should be evident in decreased time to treatment or surgical resection.

Prior to use in the clinical setting, novel diagnostic tests, including genomic assays, must undergo analytical testing to show that assay results are robust and stable under the conditions expected during routine laboratory use. This process of testing variation in the technical parameters of an assay, known as analytical validation, characterizes the performance of the test in real-life settings where a variety of reagent lots, equipment, and human operators, as well the presence of common potentially interfering agents, may be encountered [15]. Accurately estimating the nominal variation in these technical parameters is an important factor in the design of analytical variation studies.

**Fig. 1:**
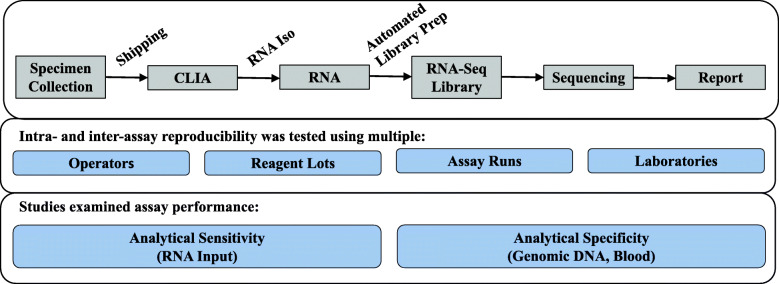
Workflow of Percepta GSC**.** Total RNA is extracted, quantified, and used as input in a library prep procedure which enriches for the coding transcriptome. After the sample is sequenced, a Percepta GSC score is calculated from an ensemble of machine learning algorithms utilizing clinical and genomic features. The Percepta GSC score is used to refine a patient’s pre-test risk stratification and a patient report is produced with the post-test risk. Assay performance was analyzed through reproducibility experiments and other studies demonstrating analytical sensitivity and analytical specificity

The Evaluation of Genomic Applications in Practice and Prevention (EGAPP) Working Group and the Centers for Disease Control’s ACCE Project (*A*nalytic validity, *C*linical validity, *C*linical utility and associated *E*thical, legal and social implications) have published criteria for evaluating the analytical validity of novel genomic tests [16, 17]. Following these criteria, Percepta GSC was evaluated for variation and reproducibility of test results in response to variation in input RNA quantity, the potentially contaminating agents blood and genomic DNA, and intra-run, inter-run, and inter-laboratory test reproducibility (Fig. 1). The results presented here demonstrate that Percepta GSC results are robust to a variety of technical variation encountered under routine laboratory testing.

The remainder of the paper is structured as follows. In the Methods section, specimen collection, laboratory workflow, and data analysis are described. In the Results section, experimental design and results of each of the analytical validation studies are described in detail. In the Discussion section, the overall robustness of Percepta GSC is discussed.

## Methods

### Specimens

Bronchial brushing samples were collected from patients who underwent bronchoscopy as part of the clinical management for suspicious lung nodules. Samples for this study were previously collected from patients enrolled in either the Airway Epithelial Gene Expression in the Diagnosis of Lung Cancer (AEGIS-1 and AEGIS-2) [11] study or the Percepta BGC Registry study [13]. Institutional review board (IRB) approval was obtained by each institution prior to study commencement and informed consent was obtained from all patients prior to their bronchoscopy. After sample collection, bronchial brushings were stored in a nucleic acid preservative (RNAprotect, QIAGEN, Hilden, Germany) and frozen at − 80 °C prior to processing.

Samples used for analytical validation were identical to those encountered in the CLIA lab in terms of sample type, quantity, collection method, and experimental process.

Fresh peripheral blood samples were collected from healthy voluntary participants. Immediately after collection into blood tubes with no preservative, blood samples were mixed with RNAprotect and subsequently processed at Veracyte.

### RNA extraction, amplification, and sequencing

Total RNA was extracted from each brushing sample according to the manufacturer’s instructions using the miRNA easy Mini Kit (QIAGEN, Hilden, Germany). Quantification was performed using the QuantiFluor RNA System (Promega, Madison, WI), and 50 ng of RNA was used as input to the TruSeq RNA Access Library Prep procedure (Illumina, San Diego, CA), which enriches for the coding transcriptome. Libraries meeting quality control criteria for amplification yields were sequenced using NextSeq 500 instruments (2 × 75 bp paired-end reads) with the High Output Kit (Illumina, San Diego, CA).

Raw sequencing (FASTQ) files were aligned to the Human Reference assembly 37 (Genome Reference Consortium) using the STAR RNA-seq aligner software [18]. Samples and sequencing runs that met pre-specified criteria for the number and quality of reads, as well as genomic representation and read depth, were used for downstream analysis. Based on annotated Ensembl genes, uniquely mapped reads were summarized using HTSeq [19]. The sequencing data was further filtered and normalized as described in [14].

### Data analysis

All data analysis was done in R version 3.5.0. The Percepta GSC is an ensemble of machine learning models using four clinical features (age, pack year, inhaled medication usage, and specimen collection timing) and 1232 gene features as inputs [14].

Linear mixed-effect models (Eq. 1) were used to evaluate the effects of RNA input amount and interfering genomic DNA on classification scores (*S*_*ijk*_). As seen in Eq. 1, *μ*_*i*_ denoted the sample effect and was modeled as a random effect, *b*_*j*_ denoted the experimental effect and was modeled as a fixed effect, *k* denoted technical replicates, and *ε*_*ijk*_ was the residual. For the total RNA input quantity analysis, the experimental effect was the RNA input amount (10, 15, 20, 36, or 50 ng RNA in one analysis and 15, 50, or 100 ng RNA in a second analysis). For the interfering genomic DNA analysis, the experimental effect was the percentage of DNA by mass added to input RNA (0, 3, 5%, or 10%). For each model, analysis of variance (ANOVA) analysis tested whether the experimental effect was significant. *P*-values were considered significant at 5%.

1 $$ {S}_{ijk}={\mu}_i+{b}_j+{\varepsilon}_{ijk} $$

To analyze assay reproducibility, the classifier scores were evaluated using linear mixed effect models (Eq. 2). For this equation, *μ*_*i*_ denoted the sample effect and was modeled as a fixed effect, *r*_*j*_ denoted the run effect and was modeled as a random effect, and *μ*_*i*_ : *r*_*j*_ denoted the interaction between sample *i* and run *j* and was modeled as a random effect.

2 $$ {S}_{ijk}={\mu}_i+{r}_j+{\mu}_i:{r}_j+{\varepsilon}_{ijk} $$

To analyze inter-laboratory accuracy, the classifier scores (*S*_*ij*_) were evaluated using a linear model (Eq. 3). In this model, *μ*_*i*_ denoted the sample effect, *k* denoted technical replicates, and *ε*_*ik*_ was the residual.

3 $$ {S}_{ik}={\mu}_i+{\varepsilon}_{ik} $$

All 95% confidence intervals (CI) for standard deviations (SD) were obtained by bootstrap where the residuals of the linear model or linear mixed-effect model were sampled with replacement.

A simulation study was performed in order to assess the degree of technical noise which could be added to classifier scores before the performance of the classifier would be severely affected. In the first step of each simulation, random score noise was generated from a normal distribution with a mean of 0 and SD varying between 0.01 and 10. This random noise was added to the Percepta GSC validation scores to create simulated scores. In the second step of each simulation, performance metrics were calculated for the simulated scores, including sensitivity, specificity, positive predictive value (PPV), and negative predictive value (NPV). The percentage of patients whose cancer risk was either increased or decreased by Percepta GSC testing was also calculated. Additionally, the flip-rate, or the percentage of altered classifier calls which occurred due to the added noise, was calculated at each noise level. The two steps described here were repeated 1000 times for each noise level, and median performance metrics were computed across all simulations at each noise level. The maximum allowable score variability was determined by choosing the largest variability for which all the performance metrics continued to meet product requirements.

## Results

### Analytical sensitivity – total RNA input quantity

**Fig. 2:**
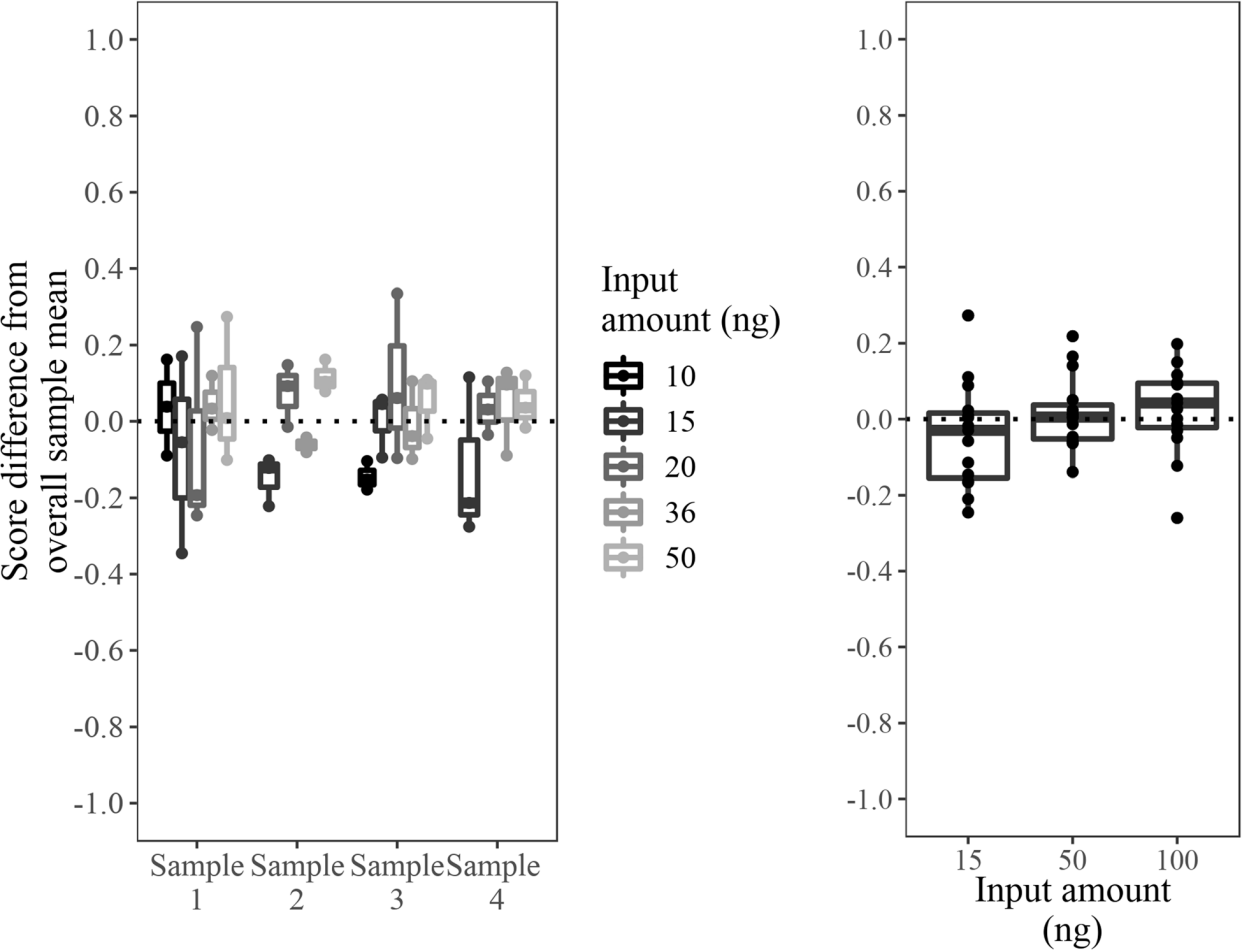
Analytical sensitivity of Percepta GSC to RNA input. The y-axis is a relative scale, with 0 representing the mean score of each sample across all input levels. In the left panel, each RNA input amount was run in triplicate for each sample. In the right panel, 15 samples were run once at each RNA input amount

The Percepta GSC test specifies that 50 ng of total RNA be used as input to the library preparation procedure, though the actual input amount may vary due to nominal quantitation measurement error (coefficients of variation (CV) of technical replicates in a single quantitation batch of up to 20%, and batch-to-batch CVs of up to 30%, data not shown) or pipetting (precision and accuracy 1% of volume typically). The sensitivity of the Percepta GSC to lower total RNA input amounts, which have the potential to result in less diverse library populations that might occur under conditions of measurement error, was evaluated. Four total RNA samples covering the Percepta GSC score space were plated in triplicate using input amounts of 10, 15, 20, 36, and 50 ng RNA, and the Percepta GSC score was determined for each run (Fig. 2). Percepta GSC scores the brushing samples were not significantly different for the lowered RNA input amounts, when evaluated with a linear mixed effect model (*p*-value = 0.07), though there was a trend towards lower Percepta GSC scores in the smallest input amount (10 ng RNA). In a separate study, 15 samples were each run once using input amounts of 15, 50 and 100 ng RNA, and the Percepta GSC scores were determined for each run (Fig. 2). Percepta GSC scores did not differ significantly with different RNA input amounts, when evaluated with a linear mixed effect model (*p*-value = 0.22). Percepta GSC test results are robust over a range beyond that which would be expected under routine test conditions, thus laboratory sample measurement error has an insignificant impact to test results even under conditions of RNA input 5-fold lower than nominal levels.

### Analytical specificity – blood

Bronchial brushing samples may contain small amounts of blood due to the procedure used to collect bronchial epithelial cells, and blood could potentially have an impact on the genomic signal [20]. The maximum theoretical blood contamination was estimated to be approximately 7.5%, based on the volume of blood that could be captured in the brush and sheath and the lowest acceptable RNA recovery [21]. Analysis of blood contamination levels in the total RNA from bronchial brush samples showed that less than 1% of the clinical samples had more than 1% of blood derived RNA, with the most extreme samples having around 10% of blood derived RNA [21]. In order to evaluate the potential of blood as an interfering substance on Percepta GSC test, whole blood total RNA from two donors was added to total RNA purified from three bronchial brushing samples. The three bronchial brushing derived RNA samples were chosen to represent the three pre-test risk groups that Percepta GSC patients are placed into by their physician prior to bronchoscopy: low (malignancy risk < 10%), intermediate (malignancy risk 10–60%), and high (malignancy risk > 60%). Pure blood-derived total RNA samples score high (malignant) in Percepta GSC score space and would theoretically increase the score for a bronchial sample when mixed. Blood samples were chosen from both the lower end and higher end of the score space seen in blood-derived total RNA samples.

Each unadulterated bronchial brushing RNA sample and blood RNA sample was run in triplicate. The two blood RNA samples were added to the three bronchial brushing derived RNA samples at 5, 10, 20, 50, and 75% blood RNA, while maintaining the total RNA input to the test constant at 50 ng. Samples admixed at the lower three proportions (5, 10, 20%) were run in triplicate, while samples admixed at higher proportions (50, 75%) were run in duplicate. Percepta GSC scores were generated for all mixtures and the best fit curve was plotted through the scores. For each bronchial sample, the percentage of added blood that would alter their original Percepta GSC was estimated from the curve (Table 1, Supplemental Figure 1).

**Table 1:** Percentage of interfering blood required to alter Percepta GSC call

Pre-Test Risk	Post-Test Risk	Low Malignant Blood	High Malignant Blood
Low	Very Low	< 28%	< 25%
	Low	> 28%	> 25%
Intermediate	Low	< 20%	< 11%
	Intermediate	> 20%	11–97%
	High	No call change	> 97%
High	Very High	No call change	No call change

With no blood RNA added, the low pre-test risk sample was reclassified to very low post-test risk. The patient’s Percepta GSC call remained unchanged compared to the pre-test risk when the amount of added blood RNA is 25% from the higher scoring blood sample or 28% from the lower scoring blood sample. With no blood RNA added, the intermediate pre-test risk sample was reclassified to a post-test risk of low risk. The patient’s Percepta GSC call remained unchanged compared to pre-test risk when the amount of added blood RNA is 11% from the higher scoring blood sample or 20% from the lower scoring blood sample. Additionally, the sample would classify as high risk at a contamination of 97% of the higher scoring blood sample. The sample did not classify as high risk at any level of contamination with the lower scoring blood sample. The pre-test high risk sample remained high post-test under all blood mixture conditions tested. Since the maximum blood content seen in patient samples was around 10%, it is unlikely that any patient calls would be expected to be influenced or changed by the presence of nominal blood contamination. Only levels of blood contamination substantially higher than thus far observed in patient samples might disrupt the accurate reclassification of samples with intermediate or low pre-test risk to low or very low post-test risk, respectively. Under these rare conditions, the utility of the test to reduce unnecessary procedures through accurate risk reduction would potentially be impacted.

### Analytical specificity – genomic DNA

**Fig. 3:**
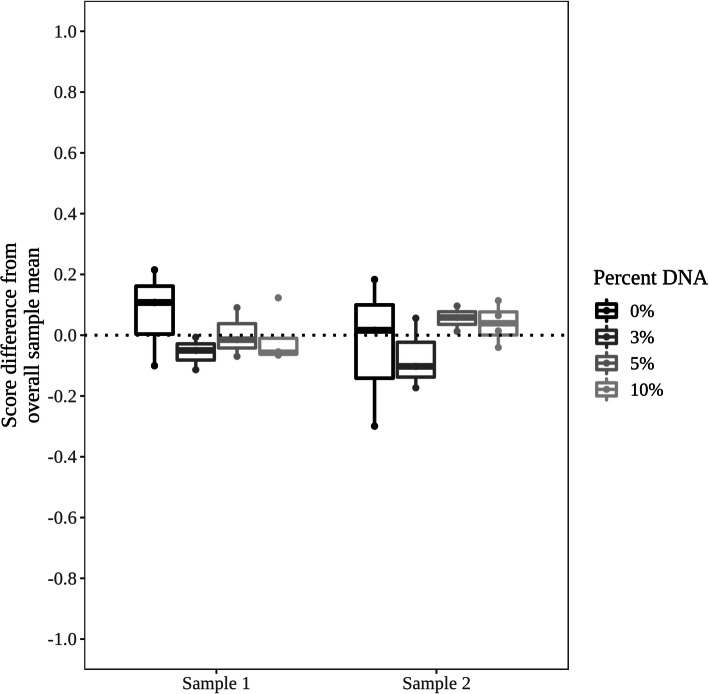
Analytical specificity of Percepta GSC against genomic DNA. The y-axis is a relative scale, with 0 representing the mean score of each sample across all input levels. Each input amount was run in triplicate at 0, 3, and 5% genomic DNA and run in quadruplicate at 10% genomic DNA. For each sample, the variation from the overall mean is plotted for each differing percentage of genomic DNA contamination

Genomic DNA is a potential contaminant and interfering substance in any biological sample, as small amounts may be co-extracted with the total RNA. In clinical samples collected as part of the AEGIS-1 and AEGIS-2 studies, the amount of genomic DNA found in the processed RNA samples was consistently 1% or less [17]. In order to test the impact of genomic DNA as a possible contaminating agent, genomic DNA was added to bronchial brushing RNA samples at 0, 3, 5, and 10% contamination by mass of nucleic acid, while maintaining the total RNA input to the test constant at 50 ng. For each amount of contamination, two samples were run in triplicate at 0, 3, and 5% genomic DNA and run in quadruplicate at 10% genomic DNA. The samples were chosen to cover Percepta GSC score space, with one high-risk scoring and one low-risk scoring sample chosen to identify any potential score shift downwards or upwards towards an intermediate post-test result, respectively. A similar analytic result (a score shift) could be detected using samples of intermediate post-test results. The Percepta GSC scores of samples with gDNA additions were not significantly different than the corresponding pure RNA samples, when evaluated with a linear mixed effect model (*p*-value = 0.49) (Fig. 3), with no consistent trend in scores observed. Therefore, Percepta GSC results are robust in the presence of DNA contamination 10-fold higher than has been observed in clinical bronchial brush samples. DNA contamination of test RNA thus has no meaningful impact to test accuracy or clinical utility.

### Assay reproducibility

**Fig. 4:**
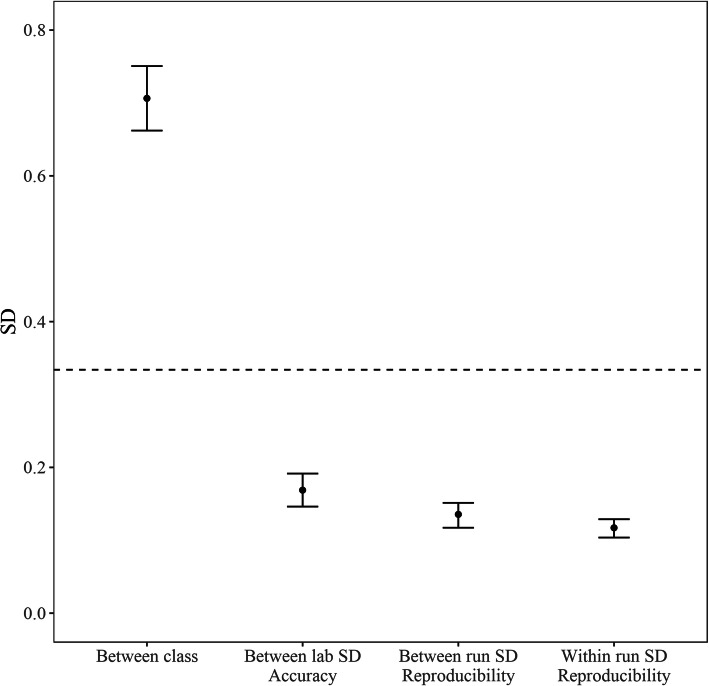
Comparison of Percepta GSC score variability. The inter-class score SD includes biological variation between cancer and benign samples and was computed from samples in the Percepta GSC validation set. The dashed line denotes the SD of 0.334, which was determined by flip-rate analysis to be the minimum SD at which performance of Percepta GSC was severely affected

In order to analyze the intra-run and inter-run reproducibility of the Percepta GSC, 33 different samples, including six control samples, were processed in triplicate across three different experimental runs in a single laboratory. For each run, the reagent lots, operators, and equipment were varied, to represent the normal variation anticipated in routine processing (Supplemental Table 1). The 27 non-control samples were chosen to represent the entire classification score space of Percepta GSC, evenly divided between high, medium, and low scoring samples. Inter-run, intra-run, and total score SDs were estimated using a mixed linear effect model. The total SD was estimated to be 0.179 (95% CI 0.169 to 0.192; Fig. 4), with the intra-run SD estimated to be 0.117 (95% CI 0.104 to 0.129; Fig. 4) and the inter-run SD estimated to be 0.136 (95% CI 0.117 to 0.151; Fig. 4). This can be compared to the inter-class score variability between benign and malignant samples, which is almost four-fold higher (Fig. 4). The total SD was compared to the score range in the training set using cross-validation, which was calculated to be 4.57. The total SD ascribed to technical variation therefore represents 3.9% of the total Percepta GSC score range. This amount of technical variation is similar to that seen in other commercial genomic classifiers (Supplemental Table 2). Thus, the technical variation within and between runs is far lower than the inherent biological signal on which the test operates.

To establish the impact of technical variability to the accuracy of the Percepta GSC, a simulation of added variability was performed in silico using 1000 simulations. Acceptable performance criteria were established for each classifier for sensitivity, specificity, NPV, and PPV, and the smallest variability at which the median performance fell below the acceptable value was determined. As technical variability was added in this simulation, the first observed effect was an increased the likelihood of a high post-test risk result for patients with intermediate pre-test risk, potentially impacting the accuracy of positive test calls. The limiting amount of variability that could be tolerated before the PPV fell below the required 50% level was 0.334, which is 7.3% of the total Percepta GSC score range. This is two-fold higher than the observed variability in the reproducibility study.

### Inter-laboratory accuracy

In order to establish the lab-to-lab accuracy of Percepta GSC, the assay was performed independently in two different laboratories: the Veracyte R&D laboratory in which the test was developed and the Veracyte reference CLIA laboratory where patient testing will be performed (Supplemental Table 1). These physically separate laboratories are operated by different personnel using different equipment. Ninety-five samples, including six control samples, were selected in order to cover the entire score space of Percepta GSC. Total SD was estimated using a linear model to be 0.169 (95% CI 0.146 to 0.192; Fig. 4), which is in line with the run to run, within lab variation observed in the reproducibility study. This suggests that technical variation associated with lab, equipment and operator variation is within the range of normal processing run variation in the same lab. The Percepta GSC scores generated between the two laboratories showed high correlation (R^2^ = 0.98). Between the two laboratories, discordant calls were made in 13 out of 131 (9.9%) classifier calls, lower than the pre-specified acceptance criteria of 15%. The discordant calls are due to normal run to run score variation in samples with scores within approximately 1 SD of the decision boundary.

## Discussion

Novel genomic tests must be characterized with respect to both clinical and analytical performance before they are put into clinical use. Potential sources of technical variation, including the entire process of sample collection, storage, shipping, handling, and laboratory processing, must be evaluated for possible impact to test results [22, 23]. Bronchial brushing specimen stability under routine collection conditions was established in the Percepta BGC analytical validation study [21]. The process described for Percepta BGC remains unchanged for clinical samples received in the Veracyte CLIA-certified laboratory for Percepta GSC testing. As such, this study was focused on analytical validation of the Percepta GSC classifier, using the new next-generation sequencing platform.

The Percepta GSC assay was found to be robust to variation in amount of RNA used in the assay and the potentially interfering substances blood and genomic DNA, with no significant difference in Percepta GSC scores noted under the conditions tested. A small impact on test performance was detected only at the extremes of RNA input (5-fold lower than nominal) or blood contamination higher than identified thus far in any bronchial brush sample. Test performance was not impacted by genomic DNA contamination at a level 10-fold higher than has been observed in clinical bronchial brush samples. To assess the impact of technical variation due to processing runs within the same laboratory versus testing done in different laboratories, Percepta GSC scores from the research laboratory where the test was developed were compared to scores generated from RNA from the same patient samples in the CLIA-certified laboratory which will run patient samples. This was in turn compared to multiple runs of the same samples within the CLIA laboratory which varied by reagent lot, equipment, day, and operator. The inter-laboratory SD of Percepta GSC scores was estimated to be 3.7% of the Percepta GSC score range, a value on par with the total SD of Percepta GSC scores within and across multiple processing runs of 3.9% of the Percepta GSC score range.

Taken together, these studies demonstrate EGAPP level I internal validity criteria for reproducibility of test results under conditions of varying operator, equipment, and process runs [16]. These results support that the Percepta GSC is a robust and stable classifier able to confidently return clinical results under routine operation in a clinical lab.

## Conclusions

Percepta GSC test results have demonstrated robustness across a variety of technical variables which may be potentially encountered as part of clinical sample testing. Use of Percepta GSC for risk stratification of suspicious lung nodules can be performed with high confidence in the clinical setting.

## Supplementary Information

**Additional file 1.**

## Data Availability

The datasets generated during the current study are not publicly available due to concerns regarding patient confidentiality and proprietary information but are available upon reasonable request from the corresponding author.

## Abbreviations

GSC: Genomic sequencing classifier
BGC: Bronchial genomic classifier
RNA: Ribonucleic acid
DNA: Deoxyribonucleic acid
EGAPP: Evaluation of genomic applications in practice and prevention
ACCE: Analytic validity, clinical validity, clinical utility and associated ethical, legal and social implications
SD: Standard deviation
CI: Confidence interval
PPV: Positive predictive value
NPV: Negative predictive value
CV: Coefficients of variation
CLIA: Clinical laboratory improvement amendments
