# Supplementary material for: Analytical validation of the Percepta genomic sequencing classifier; an RNA next generation sequencing assay for the assessment of Lung Cancer risk of suspicious pulmonary nodules
Source: BMC Cancer. 2021 Apr 13;21:400. doi: 10.1186/s12885-021-08130-x (PMC8045183; doi:10.1186/s12885-021-08130-x)
Supplement: Supplementary file 1 — Additional file 1. [file 12885_2021_8130_MOESM1_ESM.docx]

**Supplemental Figure 1.** Effect of interfering blood on Percepta GSC score. Bronchial brushing from three different pre-test risk of malignancy groups were each contaminated with RNA from two blood samples. Results from the low malignant blood sample are shown in the left panel, and the results from the high malignant blood samples are shown in the right panel. The x-axis denotes the percent of blood contamination added to each sample. For the low pre-test risk patient, the percent contamination was estimated when the curve crossed the cutoff that denoted the flip in call from intermediate to low (I/L cutoff). For the intermediate pre-test risk patient, the percent contamination was estimated when the curve crossed the cutoff that denoted the flip in call from intermediate to low (I/L cutoff) as well as the cutoff that denoted the flip in call from intermediate to high (I/H cutoff). For the high pre-test risk patient, the percent contamination was estimated when the curve crossed the cutoff that denoted the flip in call from high to very high (H/VH cutoff). Estimated values can be seen in Table 1.


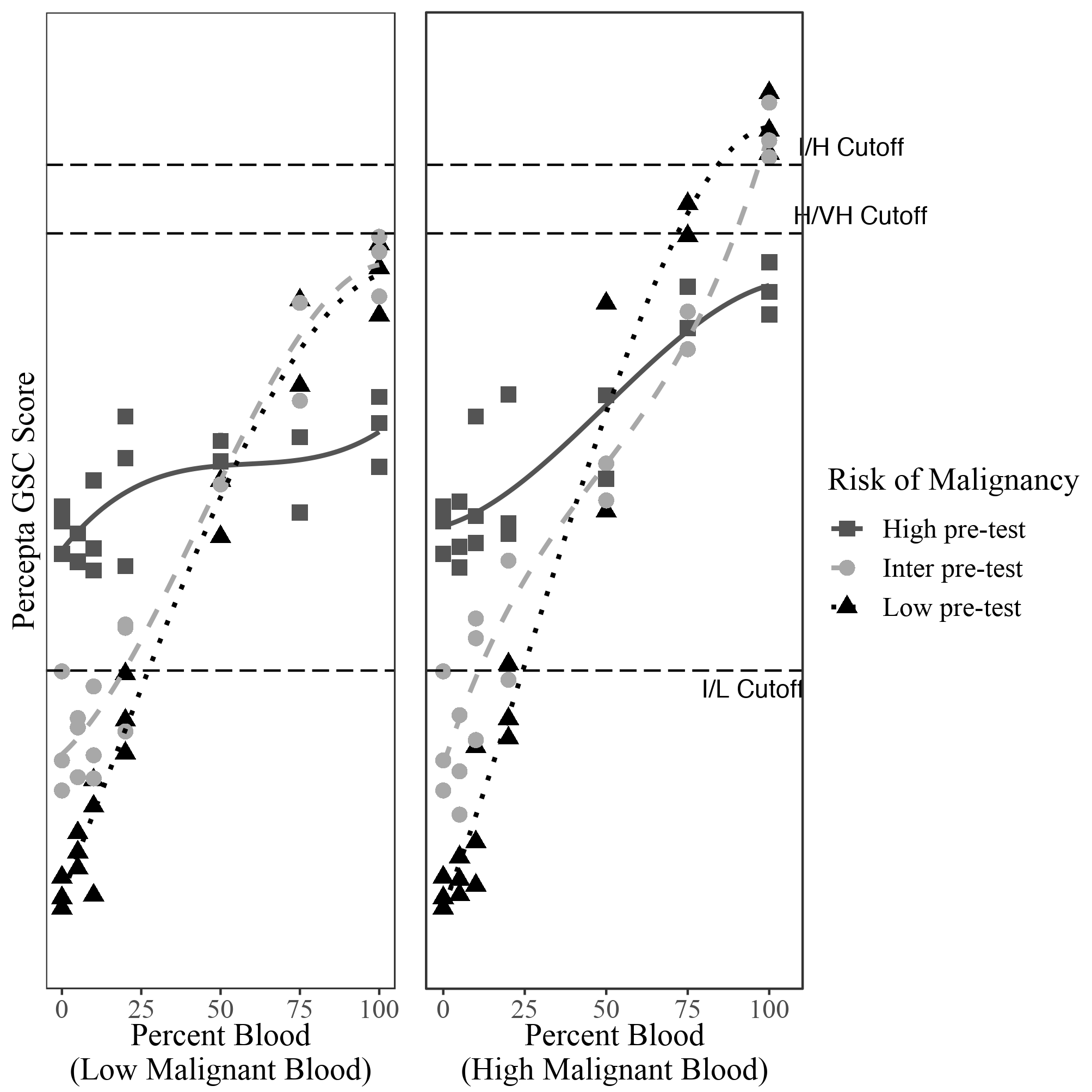


**Supplemental Table 1.** Lab, reagent lot, equipment, and operator changes made in accuracy and reproducibility plates.

|  |  | Key technical parameters | | | |
| --- | --- | --- | --- | --- | --- |
| Run | Lab | Reagent lot | Equipment pre-lab | Equipment post-lab | Operators |
| Accuracy | R&D | A117935 | A298 | B494 | MC/MW |
|  | CLIA | A117935 |  |  |  |
| Reproducibility | CLIA | A117931 | 2438 | 2403 | TS |
|  | CLIA | A117934 | 2438 | 1685 | EL |
|  | CLIA | A117936 | 2438 | 2231 | SB/TS/TT |

**Supplemental Table 2.** Variability in accuracy and reproducibility studies in commercial classifiers.

|  | SD of Accuracy Scores | SD of Reproducibility Scores | Score Range |
| --- | --- | --- | --- |
|  | (% of Score Range) | (% of Score Range) |  |
| Percepta GSC | 0.169 (3.7%) | 0.179 (3.9%) | 4.57 |
| Percepta BGC (1) | 0.276 (4.6%) | 0.199 (3.3%) | ~6 |
| Envisia (2) | 0.15 (2.1%) | 0.48 (6.9%) | ~7 |
| Afirma GSC (3) | 0.129 (1.6%) | 0.274 (3.4%) | ~8 |

**Supplemental Table 3.** List of participating sites in AEGIS and Registry study along with their IRB committee name and approval number.

| **Participating Center** | **Site Location** | **PI Name** | **IRB Name** | **IRB #** |
| --- | --- | --- | --- | --- |
| Baptist Health Louisville | Louisville, KY | Mark Esterle | Western IRB | 20151039 |
| Blount Memorial Hospital | Alcoa, TN | Gregory LeMense, Tyler Bowen | Western IRB | 20151039 |
| Central Baptist Health | Lexington, KY | Patton Thompson | Baptist Health Lexington IRB | BHL-16-1331 |
| Cooper Health | Camden, NJ | Wissam Abouzgheib | Western IRB | 20151039 |
| Duke University | Durham, NC | Momen Wahidi | DUHS IRB | Pro00064889 |
| Gundersen Clinic | La Crosse, WI | Julio Bird, Jennifer Mattingley | Gundersen Clinic Human Subjects Committee | 2-16-06-026 |
| Illinois Lung and Critical Care Institute | Peoria, IL | Patrick Whitten | University of Illinois College of Medicine at Peoria IRB | 807213 |
| Kettering Medical Center | Kettering, OH | Ehab Hussein, Hemant Shah | Western IRB | 20151039 |
| Lahey Hospital and Medical Center | Burlington, MA | Carla Lamb | Lahey Clinic IRB | 2015-064 |
| Medical College of Wisconsin | Milwaukee, WI | Ali Musani, Vijaya Ramalingam | MCW/FH IRB | Pro00025700 |
| Medical University of South Carolina | Charleston, SC | Gerard Silverstri | Medical University of South Carolina IRB | Pro00048117 |
| Memorial Health Partners | Chattanooga, TN | Krish Bhadra | Western IRB | 20151039 |
| Pinehurst Medical Center | Pinehurst, NC | Michael Pritchett | Western IRB | 20151039 |
| Pueblo Pulmonary Associates | Pueblo, CO | Joshiah Gordon | Parkview IRB | PIRB47 |
| Pulmonary Consultants | Colorado Springs, CO | Alain Eid | Western IRB | 20151039 |
| PulmonIx | Greensboro, NC | Robert Byrum | Cone Health IRB | 1993 |
| Ralph H. Johnson Veteran Affairs Medical Center | Charleston, SC | Nichole Tanner | Medical University of South Carolina IRB | Pro00048677 |
| Robert J. Dole VA | Wichita, KS | Jing Liu | KC-VAMC Human Subjects Committee | JL0009 |
| Rutgers | New Brunswick, NJ | Sugeet Jagpal | Western IRB | 20151039 |
| Schneck Medical Center | Seymour, IN | David Wilson | Western IRB | 20151039 |
| Stamford Hospital | Stamford, CT | Michael Berstein | Western IRB | 20151039 |
| Stanford University | Palo Alto, CA | Arthur Sung | Stanford University IRB | 35495 |
| The Cleveland Clinic | Cleveland, OH | Peter Mazzone | Cleveland Clinic IRB | 15-1296 |
| The Johns Hopkins Hospital | Baltimore, MD | Hans Lee | Johns Hopkins Medicine IRB | IRB00078537 |
| University of Alabama at Birmingham | Birmingham, AL | Hitesh Batra | Western IRB | 20151039 |
| University of Chicago | Chicago, IL | Kyle Hogarth, Septimiu Murgu | University of Chicago IRB | IRB15-1023 |
| University of Cincinnati | Cincinnati, OH | Sadia Benzaquen | Western IRB | 20151039 |
| University of Louisville | Louisville, KY | Tanya Wiese, Umair Gauhar | University of Louisville IRB | 15.114 |
| University of Maryland | Baltimore, MD | Ashutosh Sachdeva | UMB IRB | HP-00065862 |
| University of North Carolina | Chapel Hill, NC | Jason Akulian, Adam Belanger | Western IRB | 20151039 |
| University of Wisconsin at Madison | Madison, WI | J. Scott Ferguson | Western IRB | 20151039 |
| UT Health Athens | Tyler, TX | David Gass | UT Health East Texas IRB | 697 |
| Wake Forest Baptist Medical Center | Winston-Salem, NC | Travis Dotson | Wake Forest University IRB | IRB00034640 |
| Waterbury Pulmonary Associates | Waterbury, CT | David Hill | Western IRB | 20151039 |
| Beth Israel Deaconess Medical Center | Boston, MA | Armin Ernst , Gaetane Michaud | Beth Israel Deaconess Medical Center | 2008·P·000406/1 |
| University of British Columbia | Vancouver, BC, Canada | Stephen Lam | University of British Columbia-British Columbia Cancer Agency IRB | H08-02354 |
| University of Pennsylvania | Philadelphia, PA | Anil Vachani | University of Pennsylania IRB | 809172 |
| New York University | New York, NY | William Rom | NYU Medical Center IRB | 08-803 |
| Temple University | Philadelphia, PA | John Travaline | Temple University IRB | 12231 |
| Indiana University | Indianapolis, IN | Francis Sheski | Indiana University IRB | 1105005635 |
| University of Virginia | Charlottesville, VA | George Verghese | University of Virginia IRB for Health Sciences Research | 14315 |
| University of Missouri | Columbia, MO | Vamsi Guntur | University of Missouri Health Sciences IRB | 1128277 |
| Louisiana State University | New Orleans, LA | Stephen Kantrow | Louisiana State University Health Sciences - New Orleans IRB | 7199 |
| St. James’s Hospital, Trinity College | Dublin, Ireland | Joe Keane | SJH/AMNCH Research Ethics Committee | 031201/19303 |
| Georgia Clinical Research | Austell, GA | Stuart Simon | Western IRB | 1109725 |
| University of Wisconsin | Madison, WI | Scott Ferguson | Western IRB | 1117914 |
| Medical University of South Carolina | Charleston, SC | Gerard Silverstri | Medical University of South Carolina IRB | 20008 |
| National Jewish Health | Denver, CO | Ali Musani | Western IRB | 1119396 |
| Overlake Hospital | Bellevue, WA | Amy Markezich | Western IRB | 1118956 |
| Pulmonary Associates, P.A. | Phoenix, AZ | Mark Gotfried | Western IRB | 1120039 |
| William Jennings Bryan Dorn VAMC | Columbia, SC | Brian Smith , , rea Mass | William Jennings Brian Dorn VA Medical Center IRB | 10305 |
| Virginia Commonwealth University | Richmond, VA | Wes Shepherd | Western IRB | 1120552 |
| Jamaica Hospital Medical Center | Jamaica, NY | Craig Thurm | Jamaica Hospital Medical Center IRB | 44134 |
| University of California- Davis | Sacramento, CA | Richart Harper | University of California Davis IRB | 221372-1 |
| North Florida/South Georgia Veterans Health System | Gainesville, FL | Peruvemba Sriram | University of Florida IRB | 633-2010 |
| St. Elizabeth's Medical Center | Brighton, MA | Samaan Rafeq , Armin Ernst | St. Elizabeth's Medical Center IRB | 00554 |
| Yale | New Haven, CT | Gaetane Michaud | Yale University IRB | 1112009463 |
| Cleveland Clinic | Cleveland, OH | Tom Gildea | Cleveland Clinic IRB | 12-279 |

**References**

1. Hu Z, Whitney D, Anderson JR, Cao M, Ho C, Choi Y, Huang J, Frink R, Porta Smith K, Monroe R, Kennedy GC, Walsh PS. Analytical performance of a bronchial genomic classifier. BMC Cancer. 2016; 16:61.
2. Choi Y, Lu J, Hu Z, Pankratz DG, Jiang H, Cao M, Marchisano C, Huiras J, Fedorowicz G, Wong MG, Anderson JR, Tom EY, Babiarz J, Imtiaz U, Barth NM, Walsh PS, Kennedy GC, Huang J. Analytical performance of Envisia: a genomic classifier for usual interstitial pneumonia. BMC Pulmonary Medicine 2017 Nov 17; 17:141.
3. Hao Y, Choi Y, Babiarz JE, Kloos RT, Kennedy GC, Huang J, Walsh PS. Analytical Verificiation Performance of Afirma Genomic Sequencing Classifier in the Diagnosis of Cytologically Indeterminate Thyroid Nodules. Front. Endocrinol. 2019 Jul 4; 10:438.
